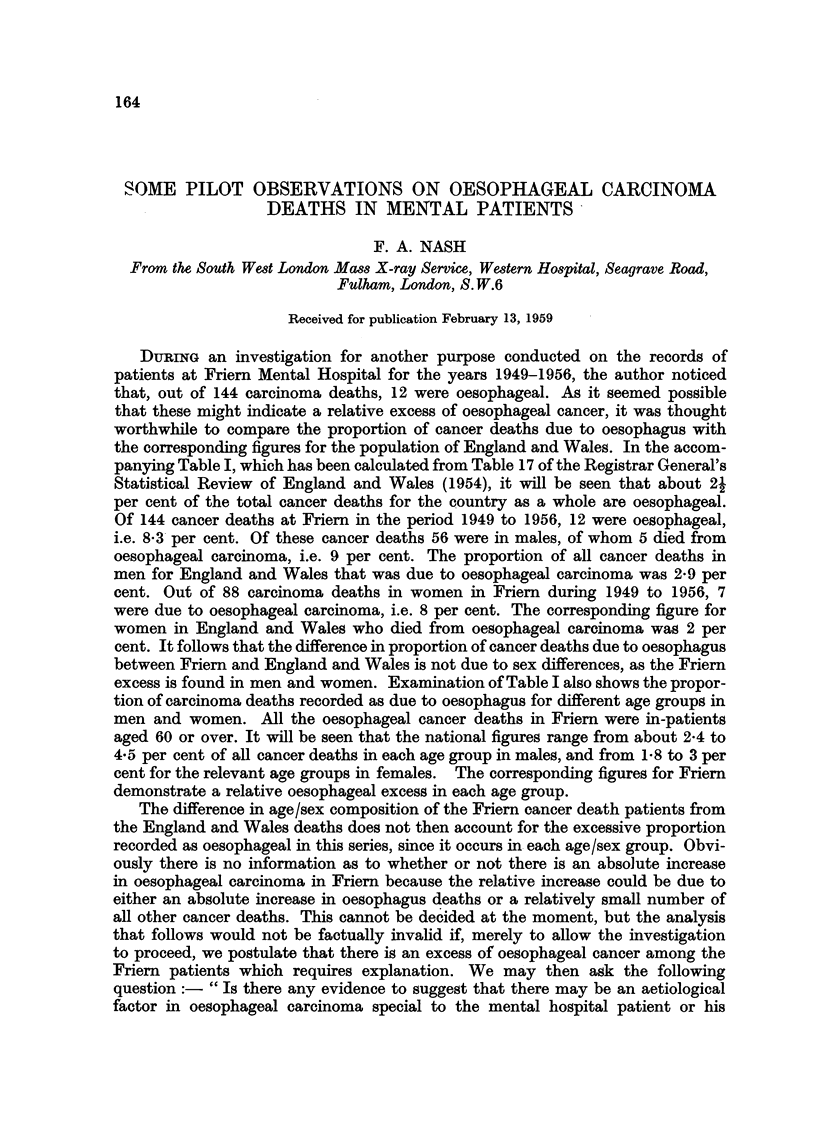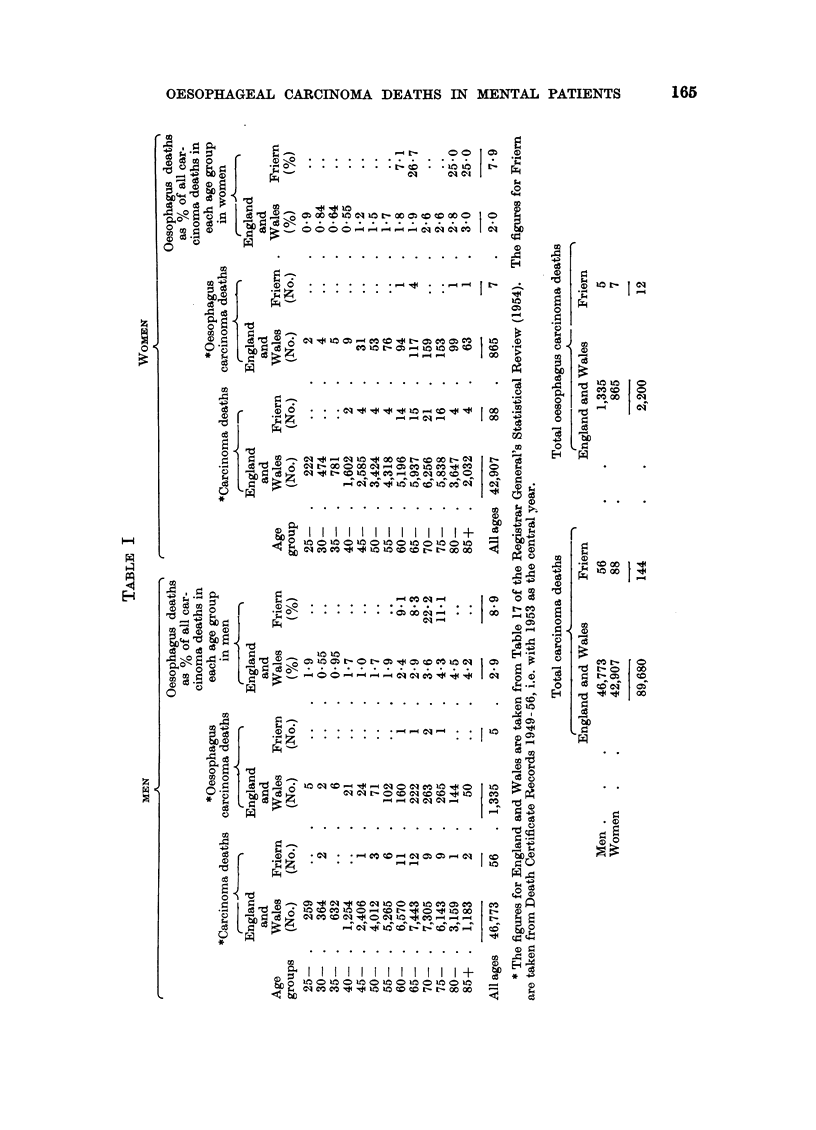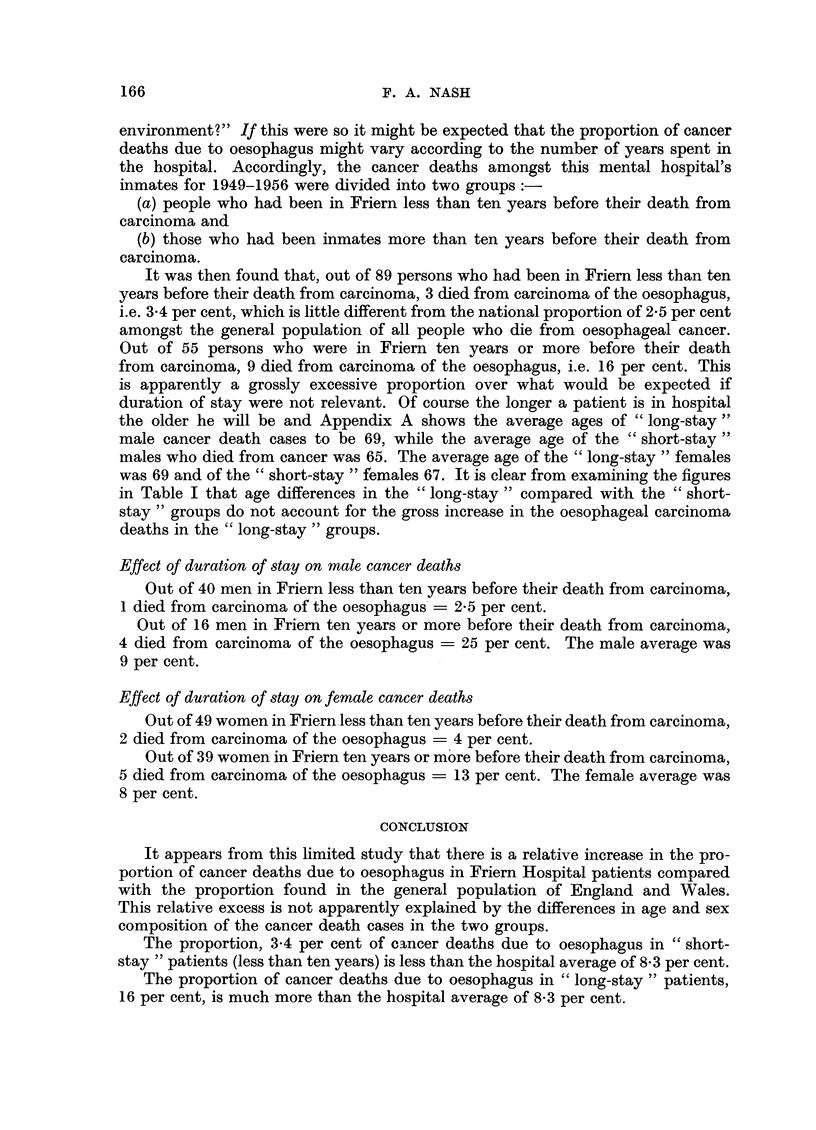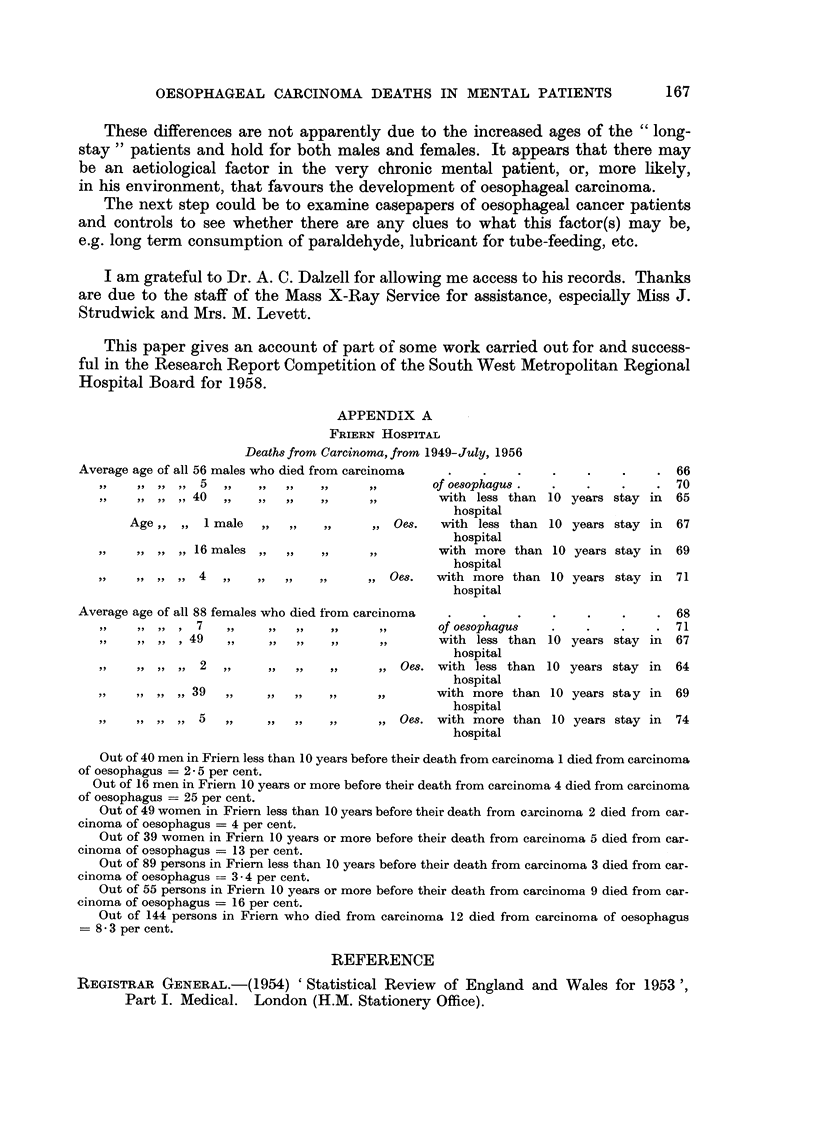# Some Pilot Observations on Oesophageal Carcinoma Deaths in Mental Patients

**DOI:** 10.1038/bjc.1959.22

**Published:** 1959-06

**Authors:** F. A. Nash


### SOME PILOT OBSERVATIONS ON OESOPHAGEAL CARCINOMA DEATHS IN MENTAL PATIENTS

F. A. NASH

*From the South West London Mass X-ray Service, Western Hospital, Seagrave Road, Fulham, London, S.W.6*

Received for publication February 13, 1959

DURING an investigation for another purpose conducted on the records of patients at Friern Mental Hospital for the years 1949–1956, the author noticed that, out of 144 carcinoma deaths, 12 were oesophageal. As it seemed possible that these might indicate a relative excess of oesophageal cancer, it was thought worthwhile to compare the proportion of cancer deaths due to oesophagus with the corresponding figures for the population of England and Wales. In the accompanying Table I, which has been calculated from Table 17 of the Registrar General's Statistical Review of England and Wales (1954), it will be seen that about $2\frac{1}{2}$ per cent of the total cancer deaths for the country as a whole are oesophageal. Of 144 cancer deaths at Friern in the period 1949 to 1956, 12 were oesophageal, i.e. 8·3 per cent. Of these cancer deaths 56 were in males, of whom 5 died from oesophageal carcinoma, i.e. 9 per cent. The proportion of all cancer deaths in men for England and Wales that was due to oesophageal carcinoma was 2·9 per cent. Out of 88 carcinoma deaths in women in Friern during 1949 to 1956, 7 were due to oesophageal carcinoma, i.e. 8 per cent. The corresponding figure for women in England and Wales who died from oesophageal carcinoma was 2 per cent. It follows that the difference in proportion of cancer deaths due to oesophagus between Friern and England and Wales is not due to sex differences, as the Friern excess is found in men and women. Examination of Table I also shows the proportion of carcinoma deaths recorded as due to oesophagus for different age groups in men and women. All the oesophageal cancer deaths in Friern were in-patients aged 60 or over. It will be seen that the national figures range from about 2·4 to 4·5 per cent of all cancer deaths in each age group in males, and from 1·8 to 3 per cent for the relevant age groups in females. The corresponding figures for Friern demonstrate a relative oesophageal excess in each age group.

The difference in age/sex composition of the Friern cancer death patients from the England and Wales deaths does not then account for the excessive proportion recorded as oesophageal in this series, since it occurs in each age/sex group. Obviously there is no information as to whether or not there is an absolute increase in oesophageal carcinoma in Friern because the relative increase could be due to either an absolute increase in oesophagus deaths or a relatively small number of all other cancer deaths. This cannot be decided at the moment, but the analysis that follows would not be factually invalid if, merely to allow the investigation to proceed, we postulate that there is an excess of oesophageal cancer among the Friern patients which requires explanation. We may then ask the following question :— " Is there any evidence to suggest that there may be an aetiological factor in oesophageal carcinoma special to the mental hospital patient or his

### Table I

| | Men | | | | | | | Women | | | | | | |
|---|---|---|---|---|---|---|---|---|---|---|---|---|---|---|
| | *Carcinoma deaths | | *Oesophagus carcinoma deaths | | Oesophagus deaths as % of all carcinoma deaths in each age group in men | | | | *Carcinoma deaths | | *Oesophagus carcinoma deaths | | Oesophagus deaths as % of all carcinoma deaths in each age group in women | |
| Age groups | England and Wales (No.) | Friern (No.) | England and Wales (No.) | Friern (No.) | England and Wales (%) | Friern (%) | | Age group | England and Wales (No.) | Friern (No.) | England and Wales (No.) | Friern (No.) | England and Wales (%) | Friern (%) |
| 25— | 259 | .. | 5 | .. | 1·9 | .. | | 25— | 222 | .. | 2 | .. | 0·9 | .. |
| 30— | 364 | 2 | 2 | .. | 0·55 | .. | | 30— | 474 | .. | 4 | .. | 0·84 | .. |
| 35— | 632 | .. | 6 | .. | 0·95 | .. | | 35— | 781 | .. | 5 | .. | 0·64 | .. |
| 40— | 1,254 | .. | 21 | .. | 1·7 | .. | | 40— | 1,602 | 2 | 9 | .. | 0·55 | .. |
| 45— | 2,406 | 1 | 24 | .. | 1·0 | .. | | 45— | 2,585 | 4 | 31 | .. | 1·2 | .. |
| 50— | 4,012 | 3 | 71 | .. | 1·7 | .. | | 50— | 3,424 | 4 | 53 | .. | 1·5 | .. |
| 55— | 5,265 | 6 | 102 | .. | 1·9 | .. | | 55— | 4,318 | 4 | 76 | .. | 1·7 | .. |
| 60— | 6,570 | 11 | 160 | 1 | 2·4 | 9·1 | | 60— | 5,196 | 14 | 94 | 1 | 1·8 | 7·1 |
| 65— | 7,443 | 12 | 222 | 1 | 2·9 | 8·3 | | 65— | 5,937 | 15 | 117 | 4 | 1·9 | 26·7 |
| 70— | 7,305 | 9 | 263 | 2 | 3·6 | 22·2 | | 70— | 6,256 | 21 | 159 | .. | 2·6 | .. |
| 75— | 6,143 | 9 | 265 | 1 | 4·3 | 11·1 | | 75— | 5,838 | 16 | 153 | .. | 2·6 | .. |
| 80— | 3,159 | 1 | 144 | .. | 4·5 | .. | | 80— | 3,647 | 4 | 99 | 1 | 2·8 | 25·0 |
| 85+ | 1,183 | 2 | 50 | .. | 4·2 | .. | | 85+ | 2,032 | 4 | 63 | 1 | 3·0 | 25·0 |
| All ages | 46,773 | 56 | 1,335 | 5 | 2·9 | 8·9 | | All ages | 42,907 | 88 | 865 | 7 | 2·0 | 7·9 |

* The figures for England and Wales are taken from Table 17 of the Registrar General's Statistical Review (1954). The figures for Friern are taken from Death Certificate Records 1949-56, i.e. with 1953 as the central year.

| | Total carcinoma deaths | | Total oesophagus carcinoma deaths | |
|---|---|---|---|---|
| | England and Wales | Friern | England and Wales | Friern |
| Men | 46,773 | 56 | 1,335 | 5 |
| Women | 42,907 | 88 | 865 | 7 |
| | 89,680 | 144 | 2,200 | 12 |

environment?" *If* this were so it might be expected that the proportion of cancer deaths due to oesophagus might vary according to the number of years spent in the hospital. Accordingly, the cancer deaths amongst this mental hospital's inmates for 1949–1956 were divided into two groups :—

(*a*) people who had been in Friern less than ten years before their death from carcinoma and

(*b*) those who had been inmates more than ten years before their death from carcinoma.

It was then found that, out of 89 persons who had been in Friern less than ten years before their death from carcinoma, 3 died from carcinoma of the oesophagus, i.e. 3·4 per cent, which is little different from the national proportion of 2·5 per cent amongst the general population of all people who die from oesophageal cancer. Out of 55 persons who were in Friern ten years or more before their death from carcinoma, 9 died from carcinoma of the oesophagus, i.e. 16 per cent. This is apparently a grossly excessive proportion over what would be expected if duration of stay were not relevant. Of course the longer a patient is in hospital the older he will be and Appendix A shows the average ages of " long-stay " male cancer death cases to be 69, while the average age of the " short-stay " males who died from cancer was 65. The average age of the " long-stay " females was 69 and of the " short-stay " females 67. It is clear from examining the figures in Table I that age differences in the " long-stay " compared with the " short-stay " groups do not account for the gross increase in the oesophageal carcinoma deaths in the " long-stay " groups.

*Effect of duration of stay on male cancer deaths*

Out of 40 men in Friern less than ten years before their death from carcinoma, 1 died from carcinoma of the oesophagus = 2·5 per cent.

Out of 16 men in Friern ten years or more before their death from carcinoma, 4 died from carcinoma of the oesophagus = 25 per cent. The male average was 9 per cent.

*Effect of duration of stay on female cancer deaths*

Out of 49 women in Friern less than ten years before their death from carcinoma, 2 died from carcinoma of the oesophagus = 4 per cent.

Out of 39 women in Friern ten years or more before their death from carcinoma, 5 died from carcinoma of the oesophagus = 13 per cent. The female average was 8 per cent.

### CONCLUSION

It appears from this limited study that there is a relative increase in the proportion of cancer deaths due to oesophagus in Friern Hospital patients compared with the proportion found in the general population of England and Wales. This relative excess is not apparently explained by the differences in age and sex composition of the cancer death cases in the two groups.

The proportion, 3·4 per cent of cancer deaths due to oesophagus in " short-stay " patients (less than ten years) is less than the hospital average of 8·3 per cent.

The proportion of cancer deaths due to oesophagus in " long-stay " patients, 16 per cent, is much more than the hospital average of 8·3 per cent.

These differences are not apparently due to the increased ages of the " long-stay " patients and hold for both males and females. It appears that there may be an aetiological factor in the very chronic mental patient, or, more likely, in his environment, that favours the development of oesophageal carcinoma.

The next step could be to examine casepapers of oesophageal cancer patients and controls to see whether there are any clues to what this factor(s) may be, e.g. long term consumption of paraldehyde, lubricant for tube-feeding, etc.

I am grateful to Dr. A. C. Dalzell for allowing me access to his records. Thanks are due to the staff of the Mass X-Ray Service for assistance, especially Miss J. Strudwick and Mrs. M. Levett.

This paper gives an account of part of some work carried out for and successful in the Research Report Competition of the South West Metropolitan Regional Hospital Board for 1958.

### APPENDIX A

### Friern Hospital

*Deaths from Carcinoma, from* 1949–*July*, 1956

| | | |
|---|---|---|
| Average age of all 56 males who died from carcinoma | . . . . . . . . . | 66 |
| ,, ,, ,, ,, 5 ,, ,, ,, ,, ,, | *of oesophagus* . . . . . | 70 |
| ,, ,, ,, ,, 40 ,, ,, ,, ,, ,, | with less than 10 years stay in hospital | 65 |
| Age ,, ,, 1 male ,, ,, ,, ,, *Oes.* | with less than 10 years stay in hospital | 67 |
| ,, ,, ,, ,, 16 males ,, ,, ,, ,, | with more than 10 years stay in hospital | 69 |
| ,, ,, ,, ,, 4 ,, ,, ,, ,, ,, *Oes.* | with more than 10 years stay in hospital | 71 |
| Average age of all 88 females who died from carcinoma | . . . . . . . . . | 68 |
| ,, ,, ,, , 7 ,, ,, ,, ,, ,, | *of oesophagus* . . . . . | 71 |
| ,, ,, ,, , 49 ,, ,, ,, ,, ,, | with less than 10 years stay in hospital | 67 |
| ,, ,, ,, ,, 2 ,, ,, ,, ,, ,, *Oes.* | with less than 10 years stay in hospital | 64 |
| ,, ,, ,, ,, 39 ,, ,, ,, ,, ,, | with more than 10 years stay in hospital | 69 |
| ,, ,, ,, ,, 5 ,, ,, ,, ,, ,, *Oes.* | with more than 10 years stay in hospital | 74 |

Out of 40 men in Friern less than 10 years before their death from carcinoma 1 died from carcinoma of oesophagus = 2·5 per cent.

Out of 16 men in Friern 10 years or more before their death from carcinoma 4 died from carcinoma of oesophagus = 25 per cent.

Out of 49 women in Friern less than 10 years before their death from carcinoma 2 died from carcinoma of oesophagus = 4 per cent.

Out of 39 women in Friern 10 years or more before their death from carcinoma 5 died from carcinoma of oesophagus = 13 per cent.

Out of 89 persons in Friern less than 10 years before their death from carcinoma 3 died from carcinoma of oesophagus = 3·4 per cent.

Out of 55 persons in Friern 10 years or more before their death from carcinoma 9 died from carcinoma of oesophagus = 16 per cent.

Out of 144 persons in Friern who died from carcinoma 12 died from carcinoma of oesophagus = 8·3 per cent.

### REFERENCE

Registrar General.—(1954) ' Statistical Review of England and Wales for 1953 ', Part I. Medical. London (H.M. Stationery Office).